# Immunosuppressive environment in response to androgen deprivation treatment in prostate cancer

**DOI:** 10.3389/fendo.2022.1055826

**Published:** 2022-11-24

**Authors:** Caipeng Qin, Jing Wang, Yiqing Du, Tao Xu

**Affiliations:** ^1^ Department of Urology, Peking University People’s Hospital, Beijing, China; ^2^ Department of Urologic Oncology, The First Affiliated Hospital of University of Science and Technology of China (USTC), Division of Life Sciences and Medicine, University of Science and Technology of China, Hefei, China

**Keywords:** prostate cancer, single cell RNAseq, ADT, immune microenvironment, immunotherapy

## Abstract

**Rationale:**

To invest the role of androgen deprivation therapy (ADT) on the tumor immune microenvironment of prostate cancer.

**Methods:**

Here we have profiled the transcriptomes of 19,227 single cells from 4 prostate tumors, including two cases who received ADT. To validated the single-cell analysis we use another group of patients receiving neoadjuvant ADT.

**Results:**

After receiving ADT treatment, the killing effect of prostate cancer immune cells on tumors is weakened, the interaction between immune cells and tumor cells is weakened, and the proportion of immunosuppressive cells Myeloid-derived suppressor cell (MDSC) and Regulatory T cells (Treg) cells increases.

**Conclusions:**

Our results highlight that ADT induces immunosuppressive in the prostate tumor microenvironment. These data have important implications for combining ADT with immunotherapy.

## Introduction

Prostate cancer is the most common malignancy in men worldwide (1), and androgen deprivation therapy (ADT) has become the standard treatment for locally advanced or metastatic prostate cancer (2, 3). In these patients, ADT forces changes in the tumor biology that result in distinct molecular profiles. Such as ADT treatment induces the up-regulated expression of Nuclear receptor coactivator 2(NCoA2), which could activate the PI3K signaling pathway to promote prostate cancer metastasis and CRPC (4). After receiving ADT treatment, the negative regulation of ZBTB46 by AR is abolished, and the up-regulated ZBTB46 transcriptionally activates the expression of SNAI1, a key initiator of epithelial-to-mesenchymal transition (EMT) (5). GRB10 is transcriptionally repressed by AR and mediates the activation of AKT to prompt the progress of prostate cancer after receive ADT (6). Future more, prostate cancer cells are surrounded by a complex tumor immune microenvironment, and tumor immune response to the ADT, remains elusive. Others have previously highlighted that ADT induces a complex immune response within the prostate tumor microenvironment (7, 8).Single-cell RNA-sequencing (scRNA-seq) technology enables the complexity of the TME to be revealed. Furthermore, scRNA-seq provides unique opportunities to assess the regulation, and interaction of individual cell, especial between the tumor cells and immune cells. In this Article, we have performed scRNA-seq on 4 prostate tumors with 2 cases receive ADT and obtained transcriptomic profiles for 11,367 cells. We have uncovered the effects of ADT on the immune microenvironment of prostate cancer.

## Results

### Single-cell RNA sequencing uncovers the cellular diversity of prostate cancer

**Figure 1:**
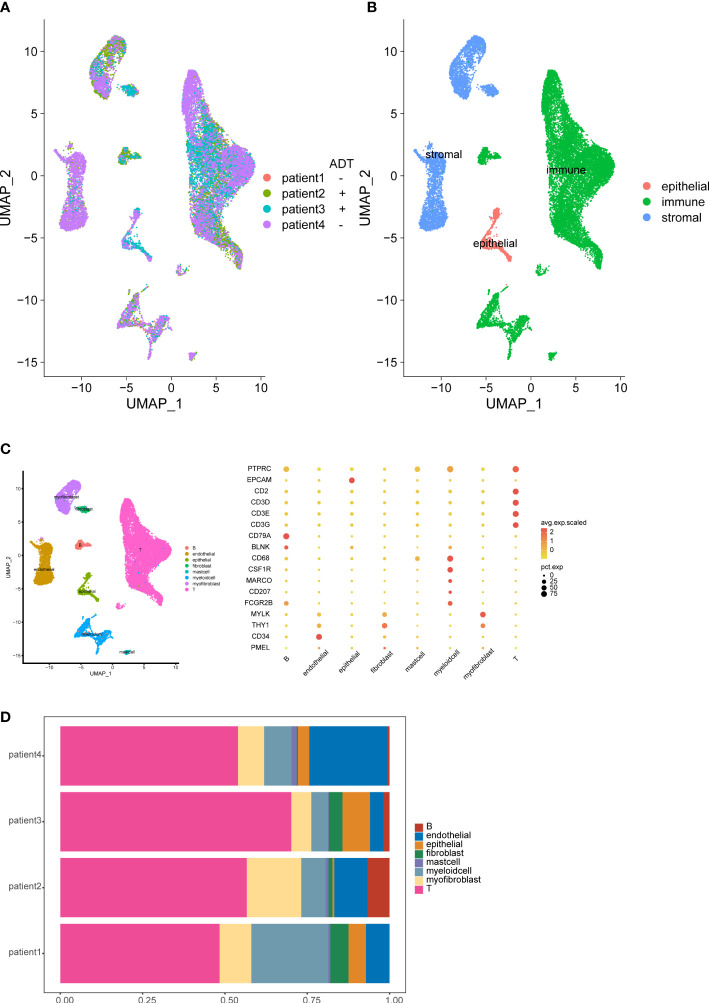
Overview of the single-cell landscape for prostate cancer. UMAPs of all single-cell transcriptomes after filtering, color-coded by patient **(A)** and quantification of main cell types per patient and UMAP of all single-cell transcriptomes color-coded by main cell type **(B)**. UMAPs of all single-cell transcriptomes color-coded by cell cluster; Marker gene expression for each cell type, where dot size and color represent percentage of marker gene expression (pct. exp) and the averaged scaled expression (avg. exp. scale) value, respectively **(C)**. Cell composition distribution for each patient sample **(D)**.

To study the cellular composition of prostate cancers, we collected 4 tissue samples from 4 patients (2 patients receive neoadjuvant ADT) and performed unsorted single-cell RNA sequencing (scRNA-seq). After standard data processing and quality control procedures, yielding high-quality transcriptomic profiles for 19,227 cells (7,570 untreated cells VS 11,657 ADT treated cells). Analysis and visualization by Uniform Manifold Approximation and Projection (UMAP) showed that single-cell transcriptomes of different patients intermingled in many clusters. Further, we analyzed the expression of immune, stromal, and epithelial marker genes to annotate the clusters. We next subset and analyzed 8 subclusters (**Figures 1A–D**).

### The tumor immune microenvironment exhibits immunosuppressive signature after ADT

**Figure 2:**
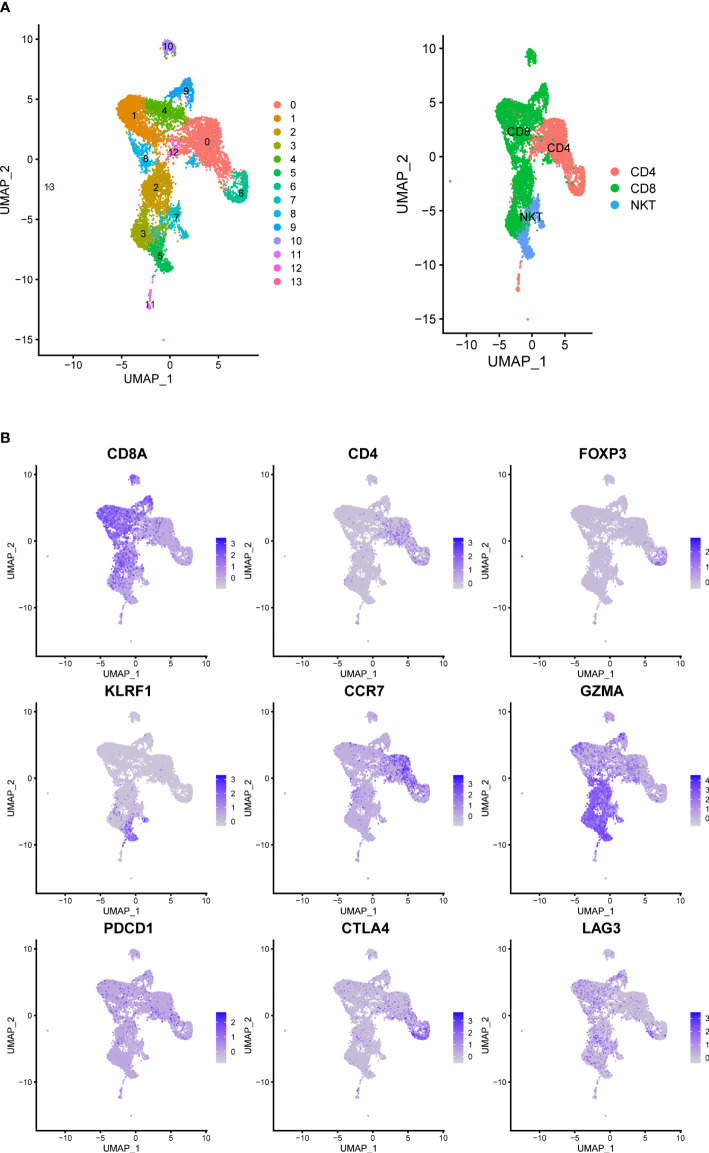
Prostate Cancer Contains Canonical T Cell. **(A)** UMAP of T-cells labeled by different cell types obtained from prostate tumors tissue with or without ADT (N = 4 patients). Phenotypic clusters are represented in distinct colors. **(B)** Relative intensity of expression of select genes superimposed on the UMAP projections.

**Figure 3:**
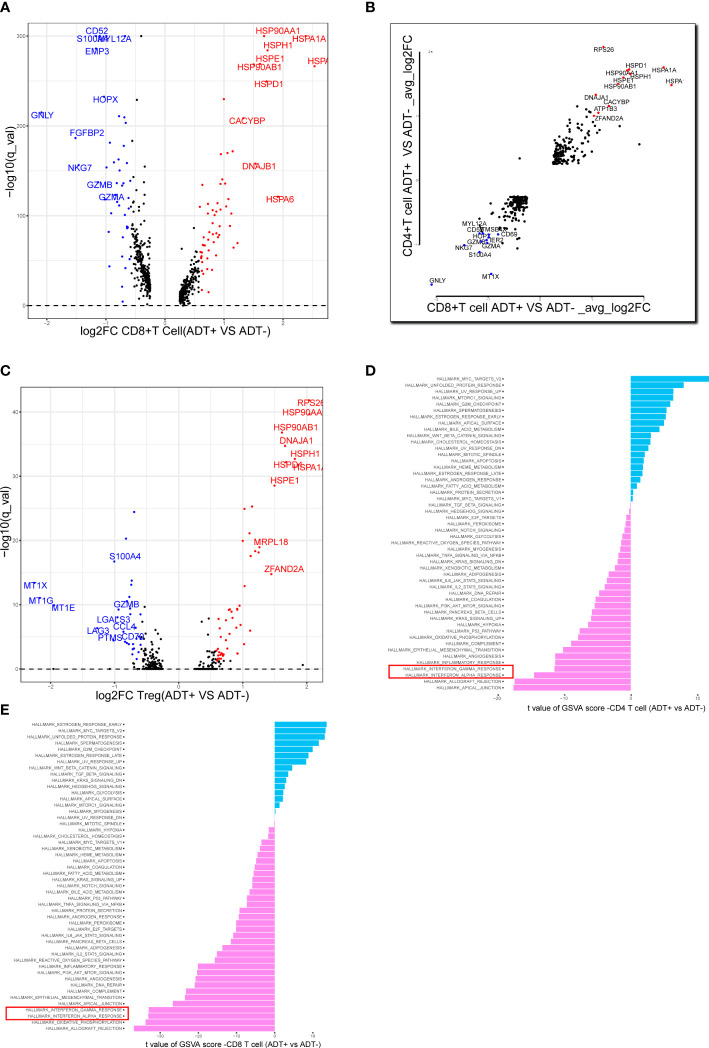
Assessing the functional states of tumor-infiltrating T cells in prostate cancer. **(A) **Volcano plot showing DEGs in ADT-derived cytotoxic T cells in comparison with those derived from without ADT tissues. Representative genes are labeled. **(B)** Scatterplot showing DEGs in ADT-derived CD8+ and CD4+ T cells in comparison with those derived from without ADT tissues. Representative genes are labeled. **(C)** Volcano plot showing DEGs in ADT-derived Treg cells in comparison with those derived from without ADT tissues. Representative genes are labeled. **(D, E)** GSVA bar plots comparing the activities of 50 hallmarks between tissue with and without ADT-derived CD4+ and CD8+ T cells.

We analyzed the gene expression of 1,1367 T cells of the prostate tumor microenvironment. We identified CD4, CD8 and NKT clusters (**Figure 2A**) based on typical marker genes (**Figures 2A, B**) and gene signatures. Cytotoxicity-related genes GNLY, NKG7, GZMB and GZMA were downregulated in both tumor-derived CD4+ and CD8+ T cells (**Figures 3A, B**). Furthermore, Regular T cells in tumors showed lower expression levels of immune regulator LGALS3, Chemokine ligands CCL4, exhaustion markers LAG3, which suggesting a suppressor immune microenvironment after ADT (**Figure 3C**). Compared with treatment naïve tissues, the gene expression results of tumor-derived CD4+ and CD8+ T cells after ADT treatment, showed downregulated in similar pathways, among which IFN response related pathways ranked high in both groups (**Figures 3D, E**). In comparison GSVA bar plots comparing the activities of 50 hallmarks between tumor-derived and nonmalignant tissue-derived CD4+ and CD8+ T cells.

**Figure 4:**
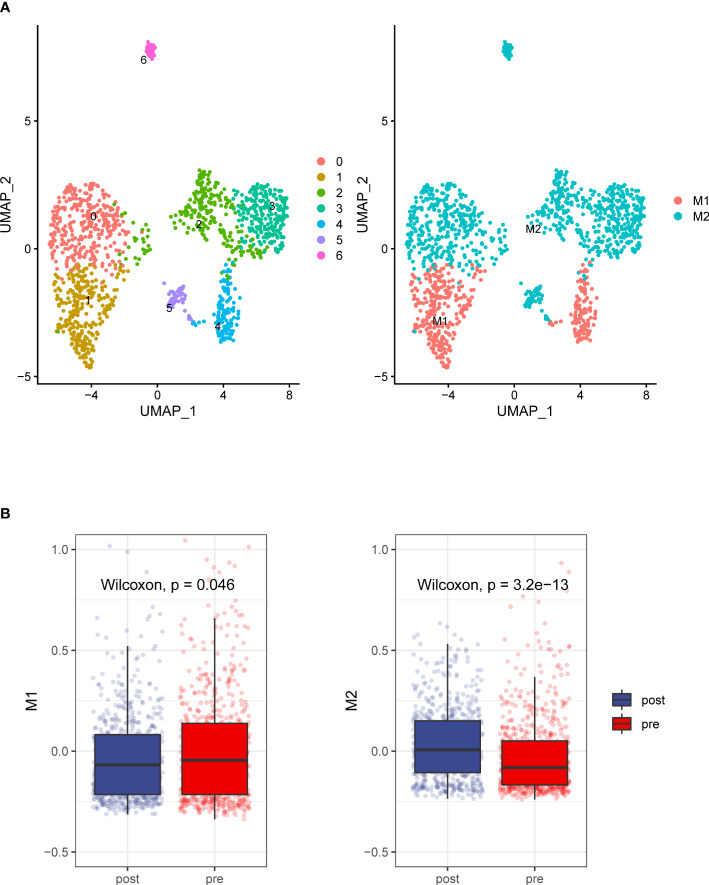
Detailed characterization of macrophage cells. **(A) **UMAP plot showing 7 clusters of macrophage cells. According to gene signature of M1 and M2, Macrophages grouped into two categories **(B)**. Compare to the prostate tumors tissue without ADT, prostate cancer tissue with ADT shows more M2 signatures and less M1 signature.

We analyzed the transcriptomes of myeloid cells, acquired 1307 macrophages based on the expression of canonical markers and their tissue origins(**Figure 4A**), We calculated an M1 score and M2 score for each cell based on previously reported markers (9). Similarly, we found that the M1 signatures weaker compared with M2 signature stronger (**Figure 4B**). This result indicates that macrophage inactivation in the tumor microenvironment (TME) after ADT.

**Figure 5:**
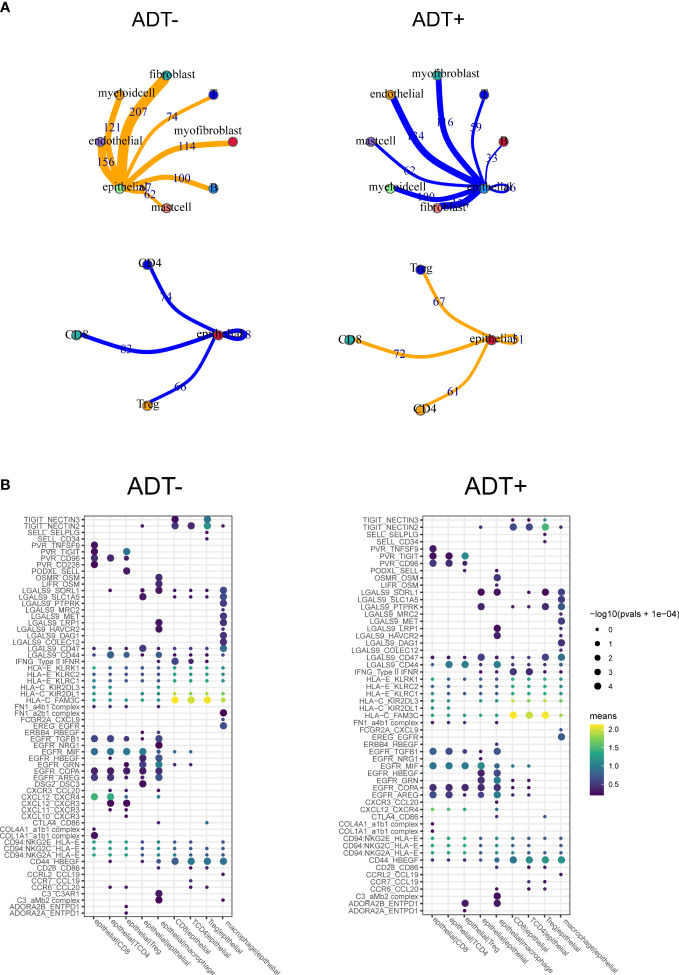
Composition and cell-cell interactions of the prostate cancer microenvironment. **(A)** View of the ligands expressed by epithelial cell and the other cells expressing the cognate receptors primed to receive the signal. Numbers indicate the quantity of ligand–receptor pairs for each intercellular link. **(B)** Summary of selected ligand-receptor interactions between epithelial cell and the other cell types. P-values (permutation test) are represented by the size of each circle. The color gradient indicates the level of interaction.

To future investigate potential influence of ADT on TME of prostate cancer, cell-cell communication analysis was performed using CellPhoneDB with or without ADT, respectively. A publicly available repository of curated receptors and ligands and their interactions is available (10). Broadcast ligands for which cognate receptors were detected demonstrated extensive communication between cells. We found immune interactions between tumor cells and T cells decreased after ADT (**Figure 5A**), including PVR-CD226, similarly, capacity to attract cytotoxic T cell (CXCL12-CXCR4) weakened (**Figure 5B**).

### ADT induces myeloid-derived suppressor cell (MDSC) infiltration and increases the distance between cytotoxic T cells and epithelial cells in the prostate cancer microenvironment

**Figure 6:**
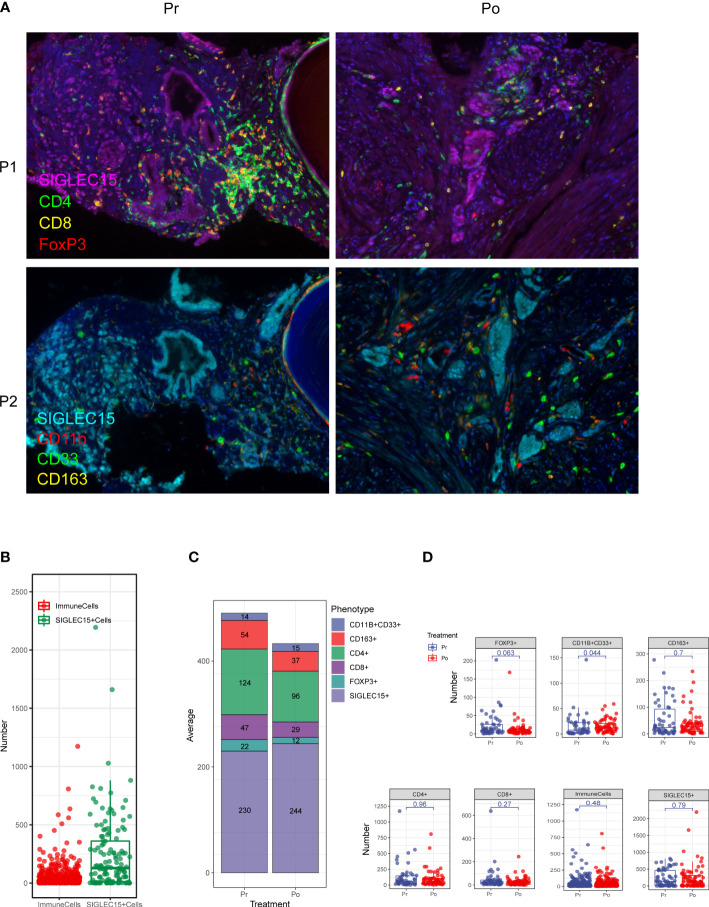
Multiplex fluorescent immunohistochemistry allows phenotyping of 6 unique cell types in the prostate cancer microenvironment. **(A)** A composite image was created incorporating all of the fluorophores present in a single tissue slide after multispectral imaging (Pr, pre-ADT; Po, post-ADT; P1, panel 1; P2, panel 2). **(B–D)** Counts of immune cells and epithelial cells per field. **(C)** The effect of ADT on the number of every cell type.

Multi-IHC staining was performed with matched pairs of pre-ADT and post-ADT samples from additional 5 patients received neoadjuvant ADT (**Figure 6A**), Siglec15 was used to marker epithelial cell, results showed significantly increased intratumoral MDSC cell density by IHC as compared to untreated matched samples (**Figures 6B–D**). MDSC may mediate prostate tumor cell immune escape.

**Figure 7:**
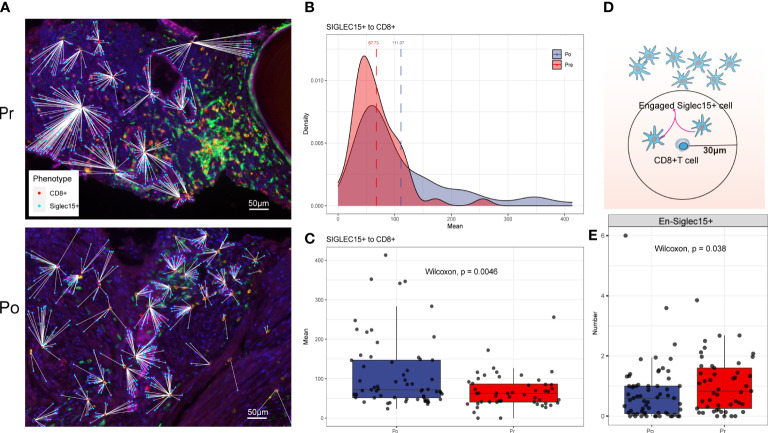
**(A–C)** ADT is associated with greater distance to cytotoxic T lymphocytes from epithelial cells. **(D, E)** ADT is associated with less cytotoxic T lymphocytes engaged epithelial cells.

Each phenotyped cells was assigned an identification number with x and y coordinates to determine their relationship to adjacent other cells (**Figure 7A**). Using R software phenoptr, the distance between epithelial cells and cytotoxic T cells were calculated for each high-power fields of tissue and the mean separation was determined, cytotoxic T cells located further away from ADT treated epithelial cells compared with naïve epithelial at 111.07μm and 67.73μm (p = 0.0046), respectively (**Figures 7B, C**).

To evaluate cellular engagement within prostate cancer environment, a circular area with a radius of 30μm was selected around each cytotoxic T cell, and the number of epithelial cells were recorded, results show that the number of engaged epithelial cell decreased after ADT (p = 0.038) (**Figures 7D, E**).

## Discussion

Typically, males are more prone to develop cancer in nearly every organ type (11). The sporadic mechanistic researches on sexual dimorphisms in cancer have been almost focused on cancer genetics (12–16). Moreover, Gonadal hormones have a significant influence on immune function (17), which may be associated with sex-biased incidence and mortality of various cancers arising in non-reproductive organs. However, Pertinent contributions by sex hormones in the tumor immune microenvironment are poorly understood.

CD8+ T cells in the tumor immune microenvironment have an enormous potential to recognize and eliminate tumor cells. On the other hand, the tumor immune microenvironment also contains cellular and molecular entities, such as regulatory T cells (18), myeloid-derived suppressor cells (19), Tumor associated macrophage (20), immune checkpoint ligands and receptors (21), inhibitory cytokines (22), and metabolic challenges (23), which promote T cell dysfunction.

Previous studies have highlighted androgen cell signal as a novel T cell intrinsic regulator prompting CD8+ T cell exhaustion, by transactivating Tcf7 to regular in CD8+ progenitor exhausted T cells (24).

Here, we try to describe the role of androgen on the prostate tumor immune microenvironment. For ADT has become the standard treatment for locally advanced or metastatic prostate cancer, but despite initial responses, recurrent castration-resistant prostate cancer (CRPC) eventually occurs (25), Multiple mechanisms have been proposed to explain the development of CRPC. The well-established dependency of cancer cells on the tumor microenvironment suggests that it might control the emergence of CRPC. Thus, our current study examines change of tumor immune microenvironment after ADT in prostate cancer.

Although ADT was associated with significantly higher levels of ICOS+ and GrB+ cells, which may represent an activated T cell subset, greater immune cell infiltration is not found in prostate tumors after ADT (8). Another study shows that ADT could lead to significant increases in intra tumoral CD8+ T cell infiltration as compared to a cohort of untreated, matched controls. However, the CD8+ T cell infiltrate is accompanied by a proportional increase in regulatory T cells (Treg), suggesting that adaptive Treg resistance may dampen the immunogenicity of ADT (26).Thus, Effects of ADT on the tumor immune microenvironment of prostate cancer remain elusive.

Previous studies have mostly used immunohistochemistry or flow cytometry to evaluate the proportion and phenotype of immune cells, With the application of single-cell transcriptome technology, a comprehensive assessment of immune cell function has become possible. To systematically survey the impact of ADT on the prostate cancer microenvironment, we perform scRNA-seq on 11,367 cells from 4 prostate cancer samples with two cases received ADT.

In our study, we compared the expression profiles of ADT treatment tissue-derived and treatment naïve tissue-derived CD8+ T cells, CD4+ T cells. T cells in ADT treatment tissue showed lower expression levels of cytotoxicity-related genes GNLY, NKG7, GZMB and GZMA (both CD4+ and CD8+ T cells), Interestingly, ADT treatment-derived Tregs showed a lower expression level of LGALS3 (27), which was reported to mediate Tregs, chemokine CCL4 and exhaustion markers LAG3. Meanwhile, the gene expression results of ADT treatment-derived CD4+ and CD8+ T cells, showed enrichment in similar pathways, among which IFN response-related pathways are significantly downregulated, which shows the immunosuppressive signature after ADT.

Finally, we investigate specific ligand-receptor interactions between tumor cells and the other cell type in the TME. We identified immune interactions between tumor cells and T cells decreasing, especially the immune promotion related pairs such as PVR-CD226 and CXCL12-CXCR4, which may mediate tumor cell immune escape despite heavy lymphocyte infiltration. On the other hand, spatial characterization of tumor environment analysis shows that CD8+T cells/Tumor cells engagement are decreased and accompanied by greater distance between CD8+T cells and tumor cells.

Much like classic M1 and M2 polarization models (28), macrophage activation in ADT tissue follows an M1-down and M2-up coupled pattern, in which M1 and M2 are discrete states, and coupled programs. This also remind the immune suppress effect of ADT on the prostate cancer microenvironment.

In our study we find that the proportion of myeloid-derived suppressor cells (MDSCs) increase in the prostate tissue after ADT. Former study identifies MDSCs as a driver of CRPC by activating the androgen receptor pathway in prostate tumor cells, promoting cell survival and proliferation in androgen deprived conditions (29).

In the present study, this study demonstrates that ADT promotes immunosuppressive environment in prostate cancer including the inhibition of killing capacity of T cell, M1-down and M2-up coupled pattern in the macrophage, proportion increasing of MDSCs and spatial characterization of immune cells.

## Material and methods

### Human specimens

The prostate cancer samples used were collected from patients who undergone radical prostatectomy at Peking University People`s Hospital. The study was approved by the Peking University people`s Hospital Institutional Review Board, and all patients provided informed consent. Fresh tissue samples were immediately dissected into fractions for (1) flash freezing, (2) fixation in 4% paraformaldehyde solution followed by paraffin embedding and (3) enzymatic digestion into single cells as described below.

### Preparation of single-cell suspension

Freshly collected tissue samples were used for single-cell isolation kept in MACS tissue storage solution (Miltenyi Biotec) until processing. Tissue samples were cut into ~1-mm cubic pieces in the RPMI-1640 medium (Invitrogen) with 10% fetal bovine serum (FBS, Gibco), and enzymatically digested with MACS tumor Dissociation Kit (Miltenyi Biotec, NO. 130-095-929) for 30~40 min at 37°C on a rotor, according to manufacturer’s instruction. The dissociated cells were subsequently passed through a 40 mm Cell-Strainer (Corning) and centrifuged at ~300–500g for 8 min. After the supernatant was removed, the pelleted cells were suspended in red blood cell lysis buffer and incubated for 2 min to lyse red blood cells. After washing twice with PBS (Invitrogen), the cell pellets were re-suspended in sorting buffer (PBS supplemented with 1% FBS).

### Single-cell 3′ mRNA sequencing library preparation

The 10x barcoding and complementary DNA (cDNA) synthesis were performed using 10x chromium 3′ scRNA-seq V2 chemistry according to the manufacturer’s instructions. Briefly, Single cells were sorted into 1.5 mL tubes (Eppendorf) and counted manually under the microscope. The concentration of single cell suspensions was adjusted to 300-350 cells/ul. Cells were loaded between 7,000 and 10,000 cells/chip position using the Chromium Single cell 30 Library, Gel Bead & Multiplex Kit and Chip Kit (103 Genomics, V2 barcoding chemistry) according to the manufacturer’s instructions. All the subsequent steps were performed following the standard manufacturer’s protocols. Purified libraries were analyzed by an Illumina Hiseq 4000 sequencer with 150-bp paired-end reads.

### Multi-color immunohistochemistry of human tissues

Multiplex immunofluorescence staining was obtained using PANO7-plex IHC kit, cat 0004100100 (Panovue, Beijing, China). Mouse anti-human Siglec15(Abcam, ab198684, 1:100), rabbit anti-human CD4(Abcam, ab133616, 1:500), mouse anti-human CD8A (CST, CST70306, 1:200), mouse anti-human FoxP3(Abcam, ab20034, 1:50), rabbit anti-human CD11b (CST, CST49420, 1:200), rabbit anti-human CD33 (Abcam, ab199432, 1:100), rabbit anti-human CD163(CST, CST93498, 1:500) were sequentially applied, followed by horseradish peroxidase-conjugated secondary antibody incubation and tyramide signal amplification.

The Mantra System (PerkinElmer, Waltham, Massachusetts, US) were used to scan the stained slides to obtain multispectral images. the scans were combined to build a single stack image.

Single-stained and unstained sections of images were used to extract the spectrum of autofluorescence of tissues and each fluorescein, respectively, which were further used to establish a spectral library required for multispectral unmixing by inform image analysis software (PerkinElmer, Waltham, Massachusetts, US). We obtained reconstructed images of sections with the autofluorescence removed by using this spectral library. For each patient, a total of 8-15 high-power fields were taken based on their tumor sizes.

## Data availability statement

The original contributions presented in the study are included in the article/supplementary material. Further inquiries can be directed to the corresponding authors.

## Ethics statement

The study was approved by the Peking University People’s Hospital Institutional Review Board, and all patients provided informed consent.

## Author contributions

Conceptualization, data curation, and writing, CQ. Software and visualization, JW. Review and editing, YD and TX. All authors contributed to the article and approved the submitted version.

## Funding

This study was supported by the Peking University Medicine Fund of Fostering Young Scholars’ Scientific (BMU2022PYB016) and Technological Innovation and the Fundamental Research Funds for the Central Universities.

## Conflict of interest

The authors declare that the research was conducted in the absence of any commercial or financial relationships that could be construed as a potential conflict of interest.

## Publisher’s note

All claims expressed in this article are solely those of the authors and do not necessarily represent those of their affiliated organizations, or those of the publisher, the editors and the reviewers. Any product that may be evaluated in this article, or claim that may be made by its manufacturer, is not guaranteed or endorsed by the publisher.
